# European project on osteoarthritis: design of a six-cohort study on the personal and societal burden of osteoarthritis in an older European population

**DOI:** 10.1186/1471-2474-14-138

**Published:** 2013-04-18

**Authors:** Suzan van der Pas, Maria Victoria Castell, Cyrus Cooper, Michael Denkinger, Elaine M Dennison, Mark H Edwards, Florian Herbolsheimer, Federica Limongi, Paul Lips, Stefania Maggi, Hans Nasell, Thorsten Nikolaus, Angel Otero, Nancy L Pedersen, Richard Peter, Mercedes Sanchez-Martinez, Laura A Schaap, Sabina Zambon, Natasja M van Schoor, Dorly JH Deeg

**Affiliations:** 1 Department of Epidemiology and Biostatistics, EMGO Institute for Health and Care Research, VU University Medical Center, Amsterdam, the Netherlands; 2 Department of Preventive Medicine and Public Health, Unit of Primary Care and Family Medicine, Faculty of Medicine, Universidad Autonoma de Madrid, Madrid, Spain; 3 MRC Lifecourse Epidemiology Unit, University of Southampton, Southampton General Hospital, Southampton, United Kingdom; 4 Bethesda Geriatric Clinic, University of Ulm, Ulm, Germany; 5 Department of Medical and Surgical Sciences, University of Padova, and National Research Council (CNR), Aging Branch, Institute of Neuroscience, Padova, Italy; 6 Department of Clinical Science and Education, Södersjukhuset, Karolinska Institutet, Stockholm, Sweden; 7 Department of Medical Epidemiology and Biostatistics, Karolinska Institutet, Stockholm, Sweden; 8 Department of Medicine, University of Padova, National Research Council, Aging Branch, Institute of Neuroscience, Padova, Italy

## Abstract

**Background:**

Osteoarthritis (OA), the most common form of arthritis, is a major contributor to functional impairment and loss of independence in older persons. The European Project on OSteoArthritis (EPOSA) is a collaborative study involving six European cohort studies on ageing. This project focuses on the personal and societal burden and its determinants of osteoarthritis. This paper describes the design of the project, and presents some descriptive analyses on selected variables across countries.

**Methods/design:**

EPOSA is an observational study including pre-harmonized data from European cohort studies (Germany, Italy, the Netherlands, Spain, Sweden and the United Kingdom) on older community-dwelling persons aged 65 to 85 years. In total, 2942 persons were included in the baseline study with a mean age of 74.2 years (SD 5.1), just over half were women (51,9%). The baseline assessment was conducted by a face-to-face interview followed by a clinical examination. Measures included physical, cognitive, psychological and social functioning, lifestyle behaviour, physical environment, wellbeing and care utilisation. The clinical examination included anthropometry, muscle strength, physical performance and OA exam. A follow-up assessment was performed 12–18 months after baseline.

**Discussion:**

The EPOSA study is the first population-based study including a clinical examination of OA, using pre-harmonized data across European countries. The EPOSA study provides a unique opportunity to study the determinants and consequences of OA in general populations of older persons, including both care-seeking and non care-seeking persons.

## Background

Osteoarthritis (OA), the most common form of arthritis, is characterized by focal areas of loss of articular cartilage within synovial joints, which are associated with hypertrophy of bone near the joints (osteophytes and subchondral bone sclerosis) and thickening of the capsule [1]. OA is a major contributor to functional impairment and loss of independence in older persons [2].

The prevalence of OA varies widely and may depend on OA definition (e.g. self-report versus clinical), site of interest, study population (patient-based or population-based) and country [3]. Among persons of 65 years and older, estimates of the prevalence of OA vary widely. Its prevalence has been estimated at 9.2% for knee OA and 7.2% for hip OA in the Netherlands [4], 32.6% for knee pain and 19.2% for hip pain in the United Kingdom [5], 29.8% for knee OA, 7.7% for hip OA, and 14.9% for hand OA in Italy [6], and 29.2% for knee OA and 18.5% for hand OA in Spain [7]. Recently, efforts to come to a consensus regarding the diagnosis of OA have lead to the European League Against Rheumatism (EULAR) recommendations [8,9]. However, these definitions are not yet standardized in ongoing cohort studies or in daily clinical practice.

The European Project on Osteoarthritis (EPOSA) studies the personal and societal burden and its determinants of OA in the ageing European population using data from population-based cohort studies across Europe. These cohorts include not only persons with severe OA who receive treatment, but also persons with OA who have not yet sought care and individuals without OA. Emphasis lies on the personal consequences (such as quality of life and social participation) and societal consequences (such as health care use) of OA. The EPOSA project focuses on the most common types of OA, namely knee, hip and hand OA. Population-based studies such as this are important as they provide data on the burden of the disease in terms of prevalence and impact on quality of life and health status, thus offering insight into the need for health care and prevention strategies.

The EPOSA study has been set up in two phases. In the first phase of EPOSA, post-harmonisation was used to enable data from existing cohort studies to be combined to explore prevalence rates and consequences of OA in Europe [10]. Our results showed that there was little evidence of correspondence of several core data collection instruments, in particular on the assessment of OA [10]. These findings reinforced the necessity of pre-harmonisation procedures in future waves of data collection in which agreement on used instruments and measurements is reached before starting a study, resulting in a pre-harmonized dataset. In the following phase of EPOSA pre-harmonisation procedures were used.

This paper aims to present the design of the EPOSA study, six pre-harmonized European population-based cohorts on ageing. Also, some descriptive analyses are presented on selected variables across countries.

### Study design and sample characteristics

The EPOSA project involves six cohort studies performed in six countries: Germany, Italy, the Netherlands, Spain, Sweden, and United Kingdom. Random samples from population-based cohorts performed in these countries are included. Per cohort, sufficient potential participants were contacted to include approximately 500 participants per country at baseline. In Italy, a new sample was drawn, with recruitment procedures and age/sex-distributions similar to those in the other studies. The age-range is 65–85 years in all cohorts except for the UK, which has an age-range of 71–79 years. This occurred because the UK sample was drawn from a birth cohort recruited over a 9-year period. The design and procedures of all six cohort studies have been approved by the Ethical Review Boards of the respective institutions.

### Study samples

Germany is represented by the University of Ulm, Institute of Epidemiology and Medical Biometry and Bethesda Geriatric Clinic. The study on Activity and Function in the Elderly in Ulm (ActiFE-Ulm) [11] is embedded in a European funded study on the prevalence of COPD and asthma (Indicators for Monitoring COPD and Asthma - IMCA) at baseline. The primary focus is physical activity (as measured by sensor technology) and the consequences of physical activity for cognitive, emotional and social functioning. Of the 615 randomly selected subjects of the ActiFE Ulm Study in the defined age range who were contacted by mail for written consent to participate in the EPOSA project, a total of 407 individuals agreed corresponding to a response rate of 66.2%. Participants were living in the greater Ulm, Neu-Ulm and Alb-Donau-Kreis areas.

Italy is represented by the National Research Council (CNR), Aging Branch, Institute of Neuroscience, Padova, and the Department of Medicine, University of Padova. The sample in Italy includes 468 out of a total of 638 invited independently dwelling subjects with a response rate of 73.4%. Participants were contacted by a letter signed also by the Principal Investigator and by their General Practitioner. Participants were living in a geographical area of northeastern Italy (Godega di Sant’Urbano) near the city of Treviso in the Veneto region, and they were randomly selected from the health district registries. The registration to receive health care is mandatory in Italy ensuring the complete coverage of the population aged 65 and older living in the sampling area.

The Netherlands is represented by the VU University Medical Center, EMGO Institute for Health and Care Research, Amsterdam. Data were used from the Longitudinal Aging Study Amsterdam (LASA) [12], an ongoing cohort study of predictors and consequences of changes in physical, cognitive, emotional and social functioning in older persons. For the current study, a total of 698 individuals were contacted by mail for written consent to participate in the EPOSA project, of which 574 (82.2%) agreed. The LASA sample was selected from population registers in 11 municipalities in the Netherlands and stratified for age, sex and level of urbanization.

Spain is represented by the Universidad Autónoma de Madrid Unit of Family Medicine and Primary Care – Department of Preventive Medicine and Public Health. The study included is Ageing in Peñagrande [13]. Participants were selected from the Register of Health District Area in the neighbourhood of Peñagrande, which is part of the Fuencarral district in Madrid. A total of 708 individuals were invited to participate, (468 from Aging in Peñagrande and 240 from a new population sample), of which 539 (76.1%) agreed. A total of 349 belonged to the Aging in Peñagrande survivals (2008 wave) [13] and 190 were new individuals elected among population belonging to Peñagrande and Cuatro-Caminos Primary Health Centers, two Centers of the same Health District Area. The organization of Spanish Health System with a register of all individuals living in the geographical area of influence of each Primary Health Center ensures that the sample is population based.

Sweden is represented by the Department of Medical Epidemiology and Biostatistics at the Karolinska Institute of Stockholm. The sample was drawn from the Swedish Twin Register [14]; background information on the participants is available through previous contacts by the Swedish Twin Registry. Of the 916 community dwelling participants, aged 65 – 85 years, living in Stockholm County who were invited by letter to participate, a total of 510 (55.7%) individuals participated.

The United Kingdom is represented by the University of Southampton, Southampton General Hospital, MRC Lifecourse Epidemiology Unit. The UK participants were recruited from the Hertfordshire Cohort Study (HCS), a population-based sample of men and women born during 1931–9 in Hertfordshire, who had previously been recruited for a study that examined the relationship between growth in infancy and the subsequent risk of common adult diseases, including osteoporosis and osteoarthritis [15]. A total of 592 individuals were invited by letter to participate, of which 444 (75.0%) agreed.

## Methods/design

### Procedure

The EPOSA data collection is considered a side-study in all participating cohorts (except in Italy, where a new sample was drawn). Data collection took place twice with about 12–18 months between baseline and the follow-up measurement. Data collection started from November 2010 to March 2011 in all the countries, and ended between September and November 2011. Participants were visited in their homes by trained interviewers, except for Germany, Italy and the Penagrande cohort in Spain, where participants were examined by a trained interviewer in a health care center and only disabled persons were visited in their home. The training of the interviewers took place as follows: a rheumatologist from the UK center visited each center to train the key interviewers who were also fluent in English. After the training, the key interviewer(s) trained other interviewers in their own center. An instruction manual of the clinical exam (including protocols for anthropometry, muscle strength, physical performance and osteoarthritis exam) was sent to all centers and also a dvd was made of the clinical OA examination. The duration of the interview was approximately one and a half hours.

At the end of the interview, six months later, and after the 1-year follow-up interview, participants were invited to complete a pain calendar on which they were asked to score per day the level of joint pain, changes in medication use, and health care utilisation for the following two weeks.

### Data collection

In the EPOSA study the same measurement instruments were used in all countries (see Table 1 for study domains and measurements). Osteoarthritis was assessed by self-report and a clinical examination of three sites: knees, hips and hands. Radiographs were obtained of knees and hips in the UK only.

**Table 1:** Summary of data collection domains

**Domain**	
	**Interview**
Demographics	Socio-demographic information
Health characteristics	Pain, stiffness, physical function (WOMAC/AUSCAN), chronic conditions, self-reported OA, joint replacements
Lifestyle characteristics	Smoking, alcohol use, physical activity (LAPAQ)
Social characteristics	Lubben Social Network Scale (LSNS), Maastricht Social Participation Profile (MSPP), Participation scale (P-scale)
Psychological characteristics & Wellbeing	Mini-Mental State Exam (MMSE), Hospital Anxiety Depression Scales (HADS), Pearlin Mastery Scale, Quality of life (EuroQoL), self-rated health
Health care utilisation & physical environment	Health care use and assistance, medication use, physical environment accessibility and usability (HACE), weather sensitivity, drive a car
	**Clinical exam**
Anthropometry	Height, weight, waist and calf circumference
Muscle strength	Hand grip strength
Physical performance	Walk test, balance test, chair stand test
Musculoskeletal	ACR classification criteria for hip, knee and hand OA
	**Pain calendar**
Pain	Joint pain score, pain medication, health care use

### Measures

#### Self-reported OA

Self-reported OA was measured by asking participants, “Do you have OA?”. If participants answered “yes”, location of OA was asked. The different sites were fingers, hand/wrist, elbows, shoulders, toes, feet, knee, hip, neck, and back. Self-reported knee, hip or hand OA was defined as present when the participant reported having OA in at least one site, knee, hip or hand (fingers or hand/wrist).

#### Clinical OA

Algorithms for clinical OA were developed based on the clinical classification criteria developed by the American College of Rheumatology (ACR) [16]. Algorithms were specified both for site specific OA (knee, hip and hand, respectively) and non-specific OA (any of these three joints).

The knee OA clinical diagnosis was based on both history and physical examination: pain in the knee was evaluated by the Western Ontario and McMaster Universities OA Index (WOMAC) pain subscale score [17], plus any 3 of: over 50 years of age, morning stiffness lasting <30 minutes evaluated by the WOMAC stiffness subscale (score from ‘mild’ to ‘extreme’); crepitus on active motion in at least one side; bony tenderness in at least one side; bony enlargement in at least one side, no palpable warmth of synovium in both knees.

The hip OA clinical diagnosis was based on both history and physical examination: pain in the hip was evaluated by the WOMAC pain subscale score, plus all of: pain associated with hip internal rotation in at least one side; morning stiffness lasting <60 minutes evaluated by the WOMAC stiffness subscale (score from ‘mild’ to ‘extreme’); and over 50 years of age.

The hand OA clinical diagnosis was based on both history and physical examination: the pain, aching or stiffness of the hand was evaluated by the Australian/Canadian OA Hand Index (AUSCAN) pain and stiffness subscale [18]; plus any 2 of: hard tissue enlargement of 2 or more of the 2nd and 3rd distal interphalangeal (DIPs), 2nd and 3rd proximal interphalangeal (PIPs), 1st carpometacarpal (CMC) joints of at least one hand; hard tissue enlargement of 2 or more DIPs of at least one hand; deformity of at least 1 of the 2nd and 3rd DIPs, 2nd and 3rd PIPs, 1st CMC joints of at least one hand. Swelling of the metacarpophalangeal (MCP) joints which is also included in the ACR classification criteria as a control to exclude rheumatic arthritis was only measured in the UK and Germany.

The WOMAC and AUSCAN are often used as indicators of pain or stiffness in the ACR classification criteria of OA [19]. The WOMAC pain subscale contains five items with regard to pain in the knee or hip: during walking on a flat surface, descending or ascending stairs, at night in bed, when sitting or lying, when standing. The AUSCAN pain subscale contains five items relating to pain experienced performing certain hand functions (at rest, gripping, lifting, turning, and squeezing objects). Both the WOMAC and AUSCAN ask about pain experienced in the past 48 hours. The WOMAC and AUSCAN responses to the five items on pain are scaled on a five point Likert scale ranging from none (0) to extreme pain (4). For both the WOMAC and AUSCAN missing values were imputed according to the user manual [17,18]. The scores were summed to get an overall pain score (range 0–20), and pain was defined by a score of 3 or more [20], also allowing inclusion of people with mild symptoms.

#### Radiographic OA

Hip x-rays consisted of a standard AP pelvis view. Knees were imaged with AP and lateral radiographs. The former were weight bearing with the patella positioned centrally and the latter were standing or supine with attendant knee flexion. From the total of 444 UK participants, 402 had knee radiographs and 394 hip radiographs. Joints were not imaged if they had previously been replaced. Grading of radiographs was performed by two investigators independently, based on scoring system according to Kellgren-Lawrence**.**

#### Joint replacements

The presence of joint replacements was assessed by asking participants if they had ever had joint replacement surgery. If participants answered “yes”, location of the joint replacement, year of joint replacement, and reason of joint replacement was elicited.

#### Demographics

Demographic data were collected on age, gender, education level and marital status. Education was measured by the highest level of education completed and categorized into “elementary school not completed”, “elementary school completed”, “vocational education/general secondary education”, and “college or university education”.

Marital status was assessed by asking whether the participants were single or never been married, married or cohabitating, divorced, widowed, registered partnership, or living apart.

#### Health characteristics

The WOMAC and AUSCAN Indices are tri-dimensional disease specific measures that assess the dimensions of pain, stiffness and physical function [17,18]. The WOMAC Index consists of 24 questions (5 pain, 2 stiffness, 17 physical function) and the AUSCAN Index consists of 15 questions (5 pain, 1 stiffness, 9 physical function). A description of the WOMAC and AUSCAN pain subscale is given above (see paragraph on clinical OA). The WOMAC stiffness subscale contains two items regarding stiffness in the knee or hip: after first awakening in the morning, and after sitting, lying or resting later in the day. The AUSCAN stiffness subscale contains one item relating to stiffness experienced with certain hand functions (after first awakening in the morning). Both the WOMAC and AUSCAN ask about stiffness experienced in the past 48 hours. The WOMAC and AUSCAN responses to the five items on stiffness are scaled on a five point Likert scale ranging from none (0) to extreme stiffness (4).

The WOMAC physical function subscale contains seventeen items relating to difficulty with knee and/or hip function experienced in the previous 48 hours. The AUSCAN physical function subscale contains nine items relating to difficulty with hand function experienced in the previous 48 hours. The WOMAC and AUSCAN responses were scaled on a five point Likert scale ranging from none (0) to extreme difficulty (4). For both the WOMAC and AUSCAN missing values were imputed according to the user manual [17,18]. For each WOMAC and AUSCAN dimension, subscale scores were normalized resulting in WOMAC and AUSCAN subscale scores, each ranging from 0 to 100, in which the three subscales are equally weighted [17,18].

Number of chronic conditions was measured through self-reported presence of the following chronic diseases or symptoms that lasted for at least three months or diseases for which the participant had been treated or followed by a physician: chronic non-specific lung disease, cardiovascular diseases, peripheral artery diseases, stroke, diabetes, cancer, and osteoporosis. If participants answered “yes” then they were asked to specify which diseases or type. Chronic conditions were evaluated as the number of diseases and multimorbidity was defined as the occurrence of 2 or more coexisting conditions.

#### Lifestyle characteristics

Lifestyle characteristics were measured by self-reports on smoking, alcohol and physical activity. Both current smoking status (never, former, current smoker) and smoking history (age when started smoking, age when stopped smoking) were assessed. Alcohol consumption was measured by frequency and amount over the past year. Physical activity was measured using the validated LASA Physical Activity Questionnaire (LAPAQ) [21]. Frequency and duration of activities over the past 2 weeks were asked for walking, cycling, gardening, light and heavy household work and a maximum of two sports. In order to calculate the daily activity, the frequency and duration were multiplied and subsequently divided by 14 days. A total physical activity score was calculated in minutes/day and kcal/day.

#### Social characteristics

The social characteristics were assessed using the Lubben’s Social Network Scale (LSNS), the Maastricht Social Participation Profile (MSPP), and the Participation scale. The six-item Lubben’s Social Network Scale (LSNS) was developed specifically for older populations [22] and assesses family (3 items) and friendship networks (3 items). Responses for questions measuring number of network contacts ranged from 0 (none) to 5 (nine or more). Total subscale scores were calculated ranging from 0 to 15. The Maastricht Social Participation Profile (MSPP) [23] measures frequency and diversity of *actual* social participation, and is based on definitions of social participation by older people with a chronic disease themselves. In this study two subscales of the MSPP were used: consumptive participation, such as attending an organised activity (6 items) and formal participation, such as volunteering work (3 items). The response categories range from 0 (‘not at all’) to 3 (‘more than twice a week’). A total score was calculated ranging from 0 to 21. The Participation scale (P-scale) measures participation restrictions in people with chronic (physical) impairments or disabilities [24]. Five items were used (from the total of eighteen items), measuring the perception of participation in helping others, taking part in recreational/social activities, being socially active, visiting others in the community, and visiting public places in the neighbourhood. The scale has a two-tier question and response format. First, a participant is asked to indicate whether they participate, more often (1), the same (2) or less often (3) in a particular aspect of participation compared to their peers. If people participate less often, they could indicate how great a problem this is to them, namely, no problem (1), small problem (2), medium problem (3) or large problem (5). The overall P-score is derived by summing the individual item scores. A higher score indicates a higher level of participation restriction.

#### Psychological characteristics and wellbeing

Cognitive function was measured by administering the Mini-Mental State Examination (MMSE) including 20 items [25]. A total score of the MMSE was calculated ranging from 0 to 30, and cognitive impairment was defined by a score of 23 or less [25]. Validated or translated versions of the MMSE were used in all centers [26-28].

Anxiety and depressive symptoms were evaluated by the Hospital Anxiety Depression Scales (HADS) [29]. HADS is a self-report questionnaire comprising 14 four-point Likert-scaled items, 7 for anxiety (HADS-A) and 7 for depression (HADS-D). The HADS measures levels of symptoms in the last week. HADS-A and HADS-D were used as categorical variables with cut-off level of 8 or more (range of 0 – 21) for presence of depression or anxiety.

Mastery was measured by means of the 7-item Pearlin Mastery Scale [30]. The questionnaire consists of seven statements such as “I have little control over the things that happen to me.” Response categories range from 1 = strongly disagree to 5 = strongly agree. The summed items range from 7 to 35, but for ease of interpretation 7 is subtracted, so the final scale ranges from 0 to 28, with higher scores indicating more mastery.

Wellbeing was evaluated by the EuroQoL and self-rated health. The EuroQoL (EQ-5D) consists of five questions, each representing one domain: mobility, self-care, usual activities, pain/discomfort, anxiety/depression [31]. The answer options differ between the questions, but can roughly be divided into: no problems, some problems, extreme problems. In addition, the participants were asked to assess their own health state on a visual analogue scale (EQ VAS). Self-rated health (SRH) was measured with the question: how is your health in general? [32]. Response categories are ‘very good’, ‘good’, ‘fair’, ‘bad’, or ‘very bad’.

#### Health care utilisation and physical environment

For health care utilisation, four types of health care services were assessed: hospitalization, primary care use, specialist services use and medication use. For hospitalization, this involved whether and how many times the participant had been hospitalized in the last year (no/yes). For primary care use, the number of visits to the general practitioner or nurse or visits received at home by these professionals during last month was assessed. Specialist care use was assessed by the number of visits in the last year to the rheumatologist, traumatologist, orthopaedic surgeon, physiotherapist, and podiatrist. Medication use was measured by asking participant which medication, prescribed by a doctor, they used during the past two weeks. The following information was asked: the brand name of the drug, the dosage (expressed as quantity per tablet or per 100 ml, and dosage form) and the number of times used per day /week/ as needed.

Home care services (formal and informal) was measured by asking whether the participant had received help at home in the last year. If the participant answered “yes”, questions were asked from whom they received the help, and the type of help (household or personal care).

Features of the physical environment were measured using the Home and Community Environment (HACE) instrument [33] and weather sensitivity. The HACE is a standardized, self-report instrument designed to assess barriers and facilitators in several environmental domains [33]. The following features of the neighbourhood environment from the HACE were examined: parks and walking areas that are easy to use; places to sit and rest at bus stops, in parks, or other places where people walk; and public transportation close to home. A question was also included on public facilities such as a daily supermarket, bus stop, post office, bank or community centre. Response categories were ‘a lot’, ‘some’, and ‘not at all’. When participants answered ‘a lot’ or ‘some’, they were asked whether they made use of the resources. Weather sensitivity was measured with the question: which weather condition(s) affects your pain the most? Multiple response categories are damp/rainy, cold, hot, or no particular weather condition. In addition, participants were asked if they drive a car.

### Clinical exam

#### Anthropometry

Height was measured to the nearest 0.001 m using a stadiometer. Weight was measured to the nearest 0.1 kg using a calibrated scale. Body Mass Index (BMI) was calculated as weight in kilograms divided by height in squared meters. Obesity was defined as present by a BMI of 30 kg/m^2^ or higher. The waist circumference was measured to the nearest 0.001 m midway between the lower rib margin and the iliac crest following a normal expiration. The calf circumference was measured to the nearest 0.001 m on the left leg with the participant standing straight, feet 20 cm apart, body weight equally distributed on both feet and at the level of the widest circumference of the calf.

#### Muscle strength

Grip strength was measured with a strain–gauged dynamometer. Participants were asked to perform two maximum force trials with each hand. To calculate the total score, the maximum values of the right and the left hand were summed, and divided by two. If only one hand could be used, the maximum value of that hand was taken.

#### Physical performance

Physical performance was measured by three individual performance tests, namely walking speed, repeated chair stands and standing balance based on the methods described by Guralnik et al. [34]. Walking speed was measured by time taken to walk 3 meters “as fast as possible but not running”. Chair stands were measured by time taken to rise five times from a chair in normal tempo, without using the hands. The standing balance test was measured by the ability to perform the tandem stand for 10 s (with one foot behind the other and the heel of the first foot directly touching the toes of the other foot). The participants’ times for walking speed and repeated chair stands were divided into country-specific quartiles (scores 1–4, participants who were unable to perform these two tests were scored 0). The tandem stand is categorized into three groups. For comparability with the other tests, these three groups received the following scores: unable (<3 seconds = 0), able to hold position for 3 to <10 seconds (2), and able to hold position for 10 seconds (4). Each test (walking speed, chair stand, and balance) is scored from 0 to 4, with a score of 0 representing inability to carry out the test, and 4 the best performance. A summary performance score was obtained by adding the scores of each test, ranging from 0 to 12.

### Pain calendar

Joint pain was assessed with a “pain calendar”. After the baseline interview, six months after the baseline interview and after the 1-year follow-up interview, participants were asked to complete a two-week calendar. Per day the participant indicated, with a score of 0 to 10, how much joint pain they had experienced and if they had used additional pain medication because of joint pain. They were contacted by telephone or letter if no calendar was returned even after a reminder. If the participant had joint pain, additional questions were asked on consultation of a general practitioner, medical specialist or whether they had surgery.

### Statistical analysis

A description of the attrition up to the EPOSA study is given, based on selected demographic and health characteristics. The EPOSA cohorts are compared with the last wave from each cohort study in the different countries. Baseline demographic and health characteristics are presented using descriptive statistics, frequency distributions and percentages. Prevalence rates of self-reported and clinical OA are computed per site and per country, and joint replacements are presented using percentages. All results are weighted except the response, age and sex.

### Weighting procedure

Sample weights were calculated to adjust for differences in age and sex distribution across country samples. The weights were calculated per sex and per five-year age category, using the following formula:

W=Nexp/Nobs,

with the Nobs being the number of persons in a specific age/sex category in the cohort, and Nexp being the number of persons in a specific age/sex category in the European population [35]. The expected number of persons was derived from the European Standard Population in 2010 [36].

## Results

Table 2 shows the recruitment and baseline characteristics of the six cohorts that were included in the EPOSA study. In total 4,040 participants were selected for recruitment, and 2,942 people aged between 65 to 85 years were interviewed (response rate = 72.8%). The baseline pain calendar was returned by 80.8% of the total sample.

**Table 2:** **Recruitment and demographic, health, lifestyle, social and psychological characteristics by country (weighted unless otherwise specified) **^1^

	**All countries**	**Germany**	**Italy**	**Netherlands**	**Spain**	**Sweden**	**UK**	**p-value**
**Sample size (unweighted)**								
Invited (n)	4,040	615	638	698	708	789	592	
Responded (N)	2,942	407	468	574	539	510	444	
Response rate (%)	72.8	66.2	73.4	82.2	76.1	64.6	75.0	
**Demographic characteristics**								
Age (mean (SD) range) (unweighted)	74.2 (5.1)	74.4 (5.0)	73.3 (5.2)	75.2 (5.7)	75.0 (5.5)	72.1 (5.0)	75.2 (2.6)	<.0001
Age (range in years) (unweighted)	65-85	65-85	65-85	65-85	65-85	65-85	71-80	
Sex (female) (n (%)) (unweighted)	1,527 (51.9)	164 (40.3)	249 (53.2)	317 (55.2)	268 (49.7)	306 (60.0)	222 (50.0)	<.0001
Married (yes) (n (%))	1,934 (63.7)	280 (67.4)	345 (72.8)	341 (57.5)	386 (68.8)	267 (51.5)	315 (69.1)	<.0001
Education (≥ secondary education) (n (%))	1,637 (55.0)	202 (50.1)	105 (22.4)	435 (74.5)	149 (27.6)	391 (76,7)	355 (79.1)	<.0001
**Health characteristics**								
BMI (Mean (SD) in kg/m^2^)	27.7 (4.4)	27.4 (4.0)	28.1 (4.6)	27.6 (4.5)	28.6 (4.3)	26.2 (4.0)	28.1 (4.6)	<.0001
Chronic diseases (≥2) (n (%))	791 (26.9)	100 (25.4)	111 (22.7)	195 (33.4)	182 (35.1)	109 (20.6)	94 (20.9)	<.0001
Physical performance score (mean (SD))	8.4 (2.7)	8.4 (2.7)	8.7 (2.2)	8.2 (2.8)	8.4 (2.9)	8.5 (2.4)	7.8 (2.9)	<.0001
**Lifestyle characteristics**								
Alcohol consumption (yes) (n (%))	2224 (74.9)	367 (90.0)	371 (78.1)	462 (80.7)	223 (38.9)	444 (88.8)	357 (79.0)	<.0001
Smoking status (n (%))								<.0001
Never	1,410 (49.5)	199 (50.5)	265 (58.5)	196 (36.9)	299 (58.5)	223 (43.2)	228 (52.8)	
Current	232 (8.5)	28 (7.0)	33 (8.2)	55 (10.5)	51 (9.2)	48 (10.1)	17 (3.7)	
Ex	1,289 (42.0)	180 (42.5)	170 (33.2)	320 (52.5)	189 (32.4)	231 (46.6)	199 (43.5)	
Physical activity score (min/day) (median [IQR])	172 [103–261]	215 [135–312]	238 [131–364]	145 [91–213]	148 [86–214]	160 [107–227]	189 [112–275]	<.0001
**Social characteristics**								
Social network								
Family (mean (SD)) (0–15)	9.2 (3.2)	9.2 (3.2)	9.9 (2.8)	9.6 (3.3)	9.0 (3.2)	8.6 (3.3)	9.0 (3.2)	<.0001
Friends (mean (SD)) (0–15)	7.6 (3.7)	8.5 (3.6)	7.1 (4.0)	7.7 (3.6)	6.1 (4.0)	8.3 (3.1)	8.5 (3.3)	<.0001
Social participation (0–21) (mean (SD))	5.4 (3.8)	7.2 (3.8)	4.3 (3.6)	4.9 (3.6)	4.6 (3.4)	5.3 (3.6)	7.2 (4.0)	<.0001
**Psychological characteristics**								
MMSE (median[IQR])	28 [26-29]	29 [28-30]	27 [25-28]	28 [27-29]	28 [26-29]	27 [26-28]	28 [25-29]	<.0001
Anxiety (≥8) (n (%))	568 (20.7)	54 (14.2)	235 (53.1)	88 (16.5)	87 (17.6)	40 (7.3)	64 (16.4)	<.0001
Depression (≥8) (n (%))	332 (11.9)	32 (7.7)	94 (20.9)	70 (12.7)	99 (19.5)	8 (1.2)	29 (7.4)	<.0001
Mastery (0–28) (mean (SD))	19.3 (5.0)	20.5 (4.1)	16.7 (4.5)	18.1 (4.5)	16.9 (4.2)	24.2 (3.9)	19.8 (4.4)	<.0001

^1^ The n is unweighted. The sample size may vary for some variables, because of missing values. Means and standard deviations (SD) and p-values for country-differences tested with ANOVA are presented for normally distributed continuous variables. Medians and Interquartile ranges (IQR) and p-values for country-differences tested with Kruskal-Wallis test are presented for skewed continuous variables. Percentages and p-values for country-differences tested with the Chi-squared were presented for categorical variables.

A comparison of participants and refusers, and those unable, was performed for age, gender, educational level, chronic conditions, MMSE and depressive symptoms (results not shown). Those who became refusers or unable were older in Sweden, Germany and Spain, and in Sweden and the Netherlands they had a lower education level. A number of health characteristics show poorer health for those who became refusers or unable in the EPOSA study. In Germany and the Netherlands, the refusers or unable had lower MMSE and more depressive symptoms. In Sweden, those who became refusers or unable, had more chronic conditions and lower MMSE.

Significant associations were found among all descriptive characteristics and the EPOSA countries. Overall, the average age of the sample was 74.2 years (SD = 5.1) and just over half were women (51.9%). The average age of the cohorts in Sweden and Italy was lower as compared with the other countries. The level of education was higher in the UK, Sweden and the Netherlands compared with Italy and Spain. In Sweden and the Netherlands the percentage married was lower compared with the other countries. With respect to the health characteristics, the BMI was the highest in Spain and the lowest in Sweden. In Sweden and the UK the percentage with 2 or more chronic conditions was the lowest. The average physical performance score was 8.4 (range 0–12), with the UK having the lowest physical performance score.

The alcohol consumption was the lowest in Spain (38.9%), compared to Germany which had the highest alcohol consumption (90.0%). In the UK only 3.7% of the participants were current smokers, while Sweden, the Netherlands and Spain had the highest prevalence of smokers (10.1%, 10.5% and 9.2% respectively). Italy had the highest physical activity score compared to the Netherlands with the lowest.

Overall, the average number of family relations was 9.2 (SD = 3.2), with little difference across countries, ranging from 9.9 in Italy to 8.6 in Sweden. The average number of friends was 7.6 (SD = 3.7), ranging from 8.5 in Germany and the UK to 6.1 in Spain. In Germany the participants had the highest social participation (M = 7.2) and in Italy the lowest (M = 4.3).

In general, participants had a high MMSE score (Median = 28). For the total sample, the level of anxiety was higher than that of depression (20.7% and 11.9% respectively). Italy had the highest anxiety and depression levels (53.1% and 20.9% respectively) and Sweden had the lowest (7.3% and 1.2% respectively). In general, participants had a high mastery (M = 19.3).

Table 3 presents the prevalence rates for self-reported and clinical knee, hip, and hand OA. The prevalence rate of self-reported OA was higher than that of clinical OA. The prevalence rates for self-reported OA varied from 21.8% for hip OA, 34.1% for knee OA, and 33.8% for hand OA. ACR criteria for clinical hip OA were satisfied in 171 participants, and the prevalence of clinical hip OA was 6.1%. The German participants had the lowest prevalence of clinical hip OA and the Italian, the highest. Of the total sample, 570 participants fulfilled the ACR classification criteria for clinical knee OA, and the prevalence of clinical knee OA was 20.2%. The highest prevalence rate for clinical knee OA was in Italy and the lowest in Germany. ACR criteria for clinical hand OA were satisfied in 482 participants, and the prevalence of clinical hand OA was 17.1%. In Italy the participants had the highest prevalence rate of clinical hand OA and in the Netherlands, the lowest. ACR criteria for any clinical knee, hip or hand OA was satisfied in 889 participants, and the prevalence for any clinical knee, hip or hand OA was 31.7%. Of those with any clinical knee, hip or hand OA, 68.8% had OA at one site, 25.1% at two sites and 6.1% at all three sites.

**Table 3:** Prevalence rate of self-reported and clinical OA and joint replacements by country (weighted)

	**All countries**	**Germany**	**Italy**	**Netherlands**	**Spain**	**Sweden**	**UK**	**p-value**
**Self-reported OA (%)**								
Knee	34.1	28.4	44.2	26.2	45.0	34.9	22.0	<.0001
Hip	21.8	7.0	35.5	19.1	24.1	27.3	11.6	<.0001
Hand	33.8	28.8	44.2	29.4	38.0	42.3	13.1	<.0001
Any knee, hip or hand	52.8	45.6	68.8	44.3	56.8	60.2	35.8	<.0001
Anywhere in the body	58.8	51.7	79.4	48.9	61.3	65.7	39.2	<.0001
**Clinical OA (%)**								
Knee	20.2	11.0	28.8	18.2	24.1	20.0	15.9	<.0001
Hip	6.1	0.8	13.8	6.7	4.4	5.0	4.7	<.0001
Hand	17.1	13.3	24.1	11.3	19.3	19.4	14.7	<.0001
Any knee, hip or hand	31.7	21.9	43.9	25.9	35.4	33.3	27.6	<.0001
**Joint replacements (%)**								
Knee replacements	3.9	3.5	3.2	2.9	4.9	3.3	6.5	ns
Hip replacements	6.4	7.7	6.7	9.2	2.4	6.5	5.7	<.001
Hand/fingers replacements	0.1	0	0	0.4	0.1	0.1	0	ns
Any joint replacements	12.3	12.1	10.6	15.2	10.2	11.6	14.2	ns

Overall, 12.3% of the participants had a joint replacement, with the Netherlands having the highest number of any joint replacements (15.2%) and Spain the lowest (10.2%). Most of these participants had either a hip (6.4%) or knee replacement (3.9%). No significant differences were found between countries for knee replacements. The Netherlands had the largest share of participants with hip replacements (9.2%) and Spain the smallest (2.4%).

## Discussion

To our best knowledge, the EPOSA study is the first population-based study using pre-harmonized data across European countries on older community-dwelling persons aged 65 to 85 years, where the ACR classification criteria have been applied to determine the prevalence of knee, hip and hand OA. This study combines data of existing cohort studies across six European countries varying in climate, socio-economic status, life style, and health care policies.

The combined set of assessments of physical, cognitive, psychological and social functioning, lifestyle behaviour, physical environment, wellbeing and care utilisation, together with a clinical examination including anthropometry, muscle strength, physical performance and OA exam will provide an extensive new resource for future studies on the personal and societal burden and its determinants of osteoarthritis. The inclusion of a pain calendar will allow the examination of variation in joint pain over time.

The EPOSA project focuses primarily on knee, hip and hand OA. Because the project is population-based and not a clinical study, it was necessary to limit the clinical exam to the most common types of OA. Overall, the prevalence of knee OA was the highest, followed by hand OA, and hip OA, for both self-reported and clinical OA. Cross-national differences are observed in prevalence rates of both self-reported and clinical OA. In Italy the prevalence rates for knee, hip, and hand clinical OA were the highest, and in Germany the prevalence rates for knee and hip clinical OA were the lowest. Radiographs of the knee and hip in the UK will enable further understanding of the association between radiological and clinical OA and the study characteristics [37].

The differences in prevalence rates may be partly due to national differences such as climate, health care, lifestyle or environmental factors [2]. Still, the specificity of the definition of OA is crucial for the prevalence rates. In Germany, an index question was used to ascertain whether participants had pain at each site (knee, hip or hand), before the specific WOMAC/AUSCAN pain questions were asked. This may have influenced the low prevalence rate of clinical OA in Germany.

The use of the population-based approach rather than a more limited clinical sample will allow generalisation of our findings to comparable populations of older adults. The project provides a unique opportunity to study both persons with mild and severe OA, and those seeking care and not seeking care. The study cohort is representative of older adults living in the community. Evidence of a responder bias was apparent for some of the six study cohorts. Such a response bias could impact the disease outcome and should be taken into account when using the EPOSA data [38].

The EPOSA study incorporates data from six population-based cohort studies in Europe, enabling cross-national comparison in OA prevalence rates and correlates. A study with such a high number of individuals using pre-harmonized data offers unique opportunities for longitudinal investigations.

## Competing interests

The authors declare that they have no competing interests.

## Authors’ contributions

SP drafted the manuscript. All authors contributed to the conception and design of the study. All authors read and corrected draft versions of the manuscript and approved of the printed version.

## Pre-publication history

The pre-publication history for this paper can be accessed here:

http://www.biomedcentral.com/1471-2474/14/138/prepub
